# Peripheral cytokine levels as novel predictors of survival in cancer patients treated with immune checkpoint inhibitors: A systematic review and meta-analysis

**DOI:** 10.3389/fimmu.2022.884592

**Published:** 2022-08-22

**Authors:** Xin-Cheng Mao, Chun-Cheng Yang, Ya-Fei Yang, Lun-Jie Yan, Zi-Niu Ding, Hui Liu, Yu-Chuan Yan, Zhao-Ru Dong, Dong-Xu Wang, Tao Li

**Affiliations:** ^1^ Department of General Surgery, Qilu Hospital, Shandong University, Jinan, China; ^2^ Department of Hepatobiliary Surgery, The Second Hospital of Shandong University, Jinan, China

**Keywords:** cytokine, cancer, cancer biomarker, immune checkpoint inhibitors, prognosis, survival analysis

## Abstract

**Background:**

Early identification of patients who will benefit from immune checkpoint inhibitors (ICIs) has recently become a hot issue in cancer immunotherapy. Peripheral cytokines are key regulators in the immune system that can induce the expression of immune checkpoint molecules; however, the association between peripheral cytokines and the efficiency of ICIs remains unclear.

**Methods:**

A systematic review was conducted in several public databases from inception through 3 February 2022 to identify studies investigating the association between peripheral cytokines (i.e., IL-1β, IL-2, IL-2RA, IL-2R, IL-4, IL-5, IL-6, IL-8, IL-10, IL-12, IL-15, IL-17, TNF-α, IFN-γ, and TGF-β) and ICI treatment. Survival data, including overall survival (OS) and/or progression-free survival (PFS), were extracted, and meta-analyses were performed.

**Results:**

Twenty-four studies were included in this analysis. The pooled results demonstrated that the pretreatment peripheral levels of IL-6 (univariate analysis: HR = 2.53, 95% CI = 2.21–2.89, *p* < 0.00001; multivariate analysis: HR = 2.21, 95% CI = 1.67–2.93, *p* < 0.00001) and IL-8 (univariate analysis: HR = 2.17, 95% CI = 1.98–2.38, *p* < 0.00001; multivariate analysis: HR = 1.88, 95% CI= 1.70–2.07, *p* < 0.00001) were significantly associated with worse OS of cancer patients receiving ICI treatment in both univariate and multivariate analysis. However, high heterogeneity was found for IL-6, which might be attributed to region, cancer type, treatment method, sample source, and detection method.

**Conclusion:**

The peripheral level of IL-8 may be used as a prognostic marker to identify patients with inferior response to ICIs. More high-quality prospective studies are warranted to assess the predictive value of peripheral cytokines for ICI treatment.

## Introduction

Immune escape plays significant roles in occurrence and development of cancer and is one of the most important hallmarks of cancer (1). Immune checkpoints, consisting of co-stimulatory and inhibitory signals, can not only modulate the immune system, but also protect tumor cells from immune killing (1, 2). Reducing the release of negative immunomodulatory factors using immune checkpoint inhibitors (ICIs), either alone or combination, can produce long-lasting antitumor effects, which is one of the standard treatments for numerous tumors (3). There are currently several ICIs approved by the Food and Drug Administration (FDA) in numerous cancer indications, including anti-PD-1, anti-PD-L1, and anti-CTLA-4 antibodies. In addition to the well-investigated molecules, a series of novel immune checkpoint molecules with the potential therapeutic value have been introduced, such as lymphocyte activation gene-3 (LAG-3), T-cell immunoglobulin and mucin-domain containing-3 (TIM-3), and T-cell immunoglobulin and ITIM domain (TIGIT), and monoclonal antibody drugs designed against these novel sites have shown promising curative effects (3, 4).

However, the response rate of ICI treatment varies depending on the cancer type, which is stable at 10%–40%, and most patients progress despite initial response (3, 5). On the other hand, ICI treatment is accompanied by some immune-related adverse events (irAEs), which can be serious or even fatal (6). To avoid ineffective therapies and severe irAEs, early identification of patients who will not respond to ICI treatment has recently become a hot issue in cancer therapy. Diverse predictive biomarkers associated with the response of ICIs include intratumoral expression of PD-L1, tumor mutational burden (TMB), and T-cell infiltration metrics (7). However, it is difficult to establish uniform criteria to quantity these markers, and even more difficult to collect tumor sample before therapy begins; to date, only intratumoral PD-L1 detection has received regulatory companion diagnostic approval for ICI treatment (8). Therefore, it is critical to find new prognostic markers to improve the outcome of cancer patients treated with ICIs.

Cytokines are pleiotropic regulators that can play important roles in controlling cell development, growth, survival, and differentiation, through autocrine or paracrine pathways (9). In the tumor microenvironment (TME), the main types of cytokines involved in intercellular communications include interleukins (ILs), interferons (IFNs), the tumor necrosis factor (TNF) superfamily, chemokines, and growth factors (10). In addition, cytokines can recruit more immune cells into the TME and induce the expression of immune checkpoint proteins, such as PD-1 or TIM-3, to assist tumor cells evading the immune system (11, 12). It has been suggested that serum concentrations of TGF-β and IL-10 are associated with therapeutic effect and higher levels of IL-17 can predict irAEs in melanoma patients treated with ipilimumab (13). In view of its non-invasive and accessible nature, peripheral cytokines may have potential value as prognostic markers of cancer patients treated with ICIs. However, to our knowledge, there is currently no statistical evidence to confirm this hypothesis. Therefore, we performed a comprehensive meta-analysis of relevant clinical trials to investigate the association between peripheral cytokines and the efficacy of ICI treatment in cancer patients.

## Methods

The Preferred Reporting Items for Systematic Reviews and Meta-Analyses (PRISMA) guidelines were utilized to conduct this systematic review and meta-analysis (14).

### Information sources and search strategy

A systematic retrieve was conducted in the PubMed, Embase, and Cochrane Library databases from inception through 3 February 2022 to identify articles investigating the relationship between peripheral cytokines and the efficacy of ICI treatment in cancer patients. The search strategy consisted of four major components: (1) “cancer” and synonyms; (2) “prognosis” and synonyms; (3) “immune checkpoint inhibitor” and synonyms; and (4) “cytokine” and synonyms. The search included only studies published in English and human studies. The detailed search strategy for PubMed is shown in **Supplementary Table 1**.

### Inclusion and exclusion criteria

Studies were screened regarding the following inclusion criteria: (1) patients suffering from any malignant tumors diagnosed by pathologic features or clinical characteristics; (2) treatment option including at least one ICI approved by FDA; (3) any kind of assessment of peripheral cytokines before treatment, mainly including IL-1β, IL-2, IL-2RA, IL-2R, IL-4, IL-5, IL-6, IL-8, IL-10, IL-12, IL-15, IL-17, TNF-α, IFN-γ, and TGF-β (the above types of cytokines were all identified by referring the research of Briukhovetska et al. (15) and all played critical roles in the immune process); and (4) survival analysis contained subgroup comparisons of overall survival (OS) or progression-free survival (PFS) based on concentrations of peripheral cytokine using a hazard ratio (HR). Exclusive criteria include the following: (1) studies involved therapies other than ICIs (e.g., targeted therapy, chemotherapy, radiation therapy, and other immunotherapy); (2) studies reported the serum protein levels that were not related to cytokine; and (3) case report, review, letter, and conference abstract.

### Data extraction and quality assessment

Two investigators (MXC and LH) independently extracted HR and 95% confidence interval (CI) from each included studies in addition to methodological and patient characteristics. The Parmar method was used to extract survival data when studies did not provide the original data.

The two investigators (MXC and LH) also independently reviewed the included studies to assess their methodological quality by using Newcastle–Ottawa scale (NOS) score, which ranged from 0 to 9. Studies with scores ≥5 were defined as high-quality studies. Any disagreements were resolved by discussing and receiving a consensus with a third author (LT).

### Statistical analysis

Statistical analysis was conducted only when each cytokine contains at least three individual survival data. OS and/or PFS for each cytokine were calculated, providing a pooled HR. The between-study heterogeneity was measured applying *I* ^2^ test with the *p*-value. Significant heterogeneity was suggested if *I* ^2^ > 50%, and a random-effects model was used to summary data. Otherwise, we used a fixed-effects model. Publication bias was assessed inspecting funnel plot asymmetry and examined using Egger’s test. A *p*-value <0.1 was considered the presence of publication bias. Sensitivity analyses were conducted for statistically significant HR by omitting each of the included studies in turn to verify the stability of the pooled results. To further investigate sources of the between-study heterogeneity, subgroup analysis and meta-regression analysis were conducted. The following potential confounders were considered: region, cancer type, sample source, detection time, treatment, and detection method. All analyses were performed using Stata and R software. *p*-value ≤0.05 was considered statistically significant.

## Results

### Characteristics of included studies

**Figure 1:**
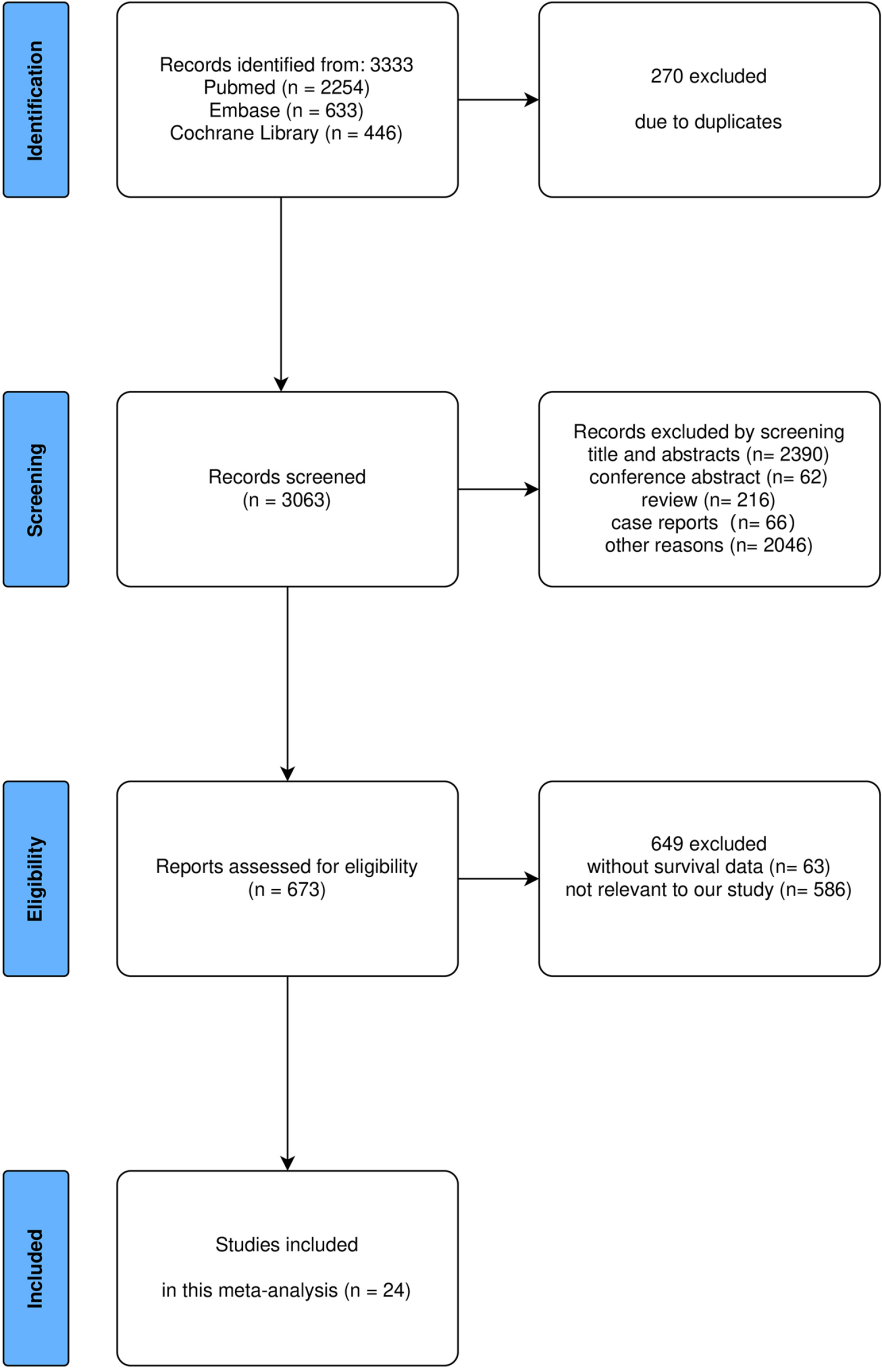
Flowchart of study selection.

In total, 3,333 records were identified, of which 24 studies met the inclusion criteria and were eligible for this meta-analysis (7, 13, 16–37) (**Figure 1**). The 24 studies utilized 44 datasets to produce their analyses (4 studies utilized 2 datasets, 1 study utilized 5 datasets, 1 study utilized 6 datasets, and 1 study utilized 7 datasets). The characteristics of the 24 included studies are summarized in **Table 1**. These studies were conducted in 11 countries. Nine studies analyzed patients with melanoma, eight studies analyzed patients with lung cancer, two studies analyzed patients with esophageal cancer, one study analyzed patients with prostate cancer, one study analyzed patients with head and neck cancer, and of the rest, three studies included multiple cancer types. A total of 23 studies adopted ICI monotherapy, and 1 study adopted combination ICI plus chemotherapy strategy. The number of patients included in these studies ranged from 16 to 1,642. The NOS scores among included studies ranged from 6 to 10 points.

**Table 1:** Study characteristics and quality assessment.

No.	Study	Sample size (*n*)	Male (%)	Treatment	Cancer type	Markers measured	Quality rating
1	Zhou et al., 2021, (16) China	156	89.1	Anti-PD-1; Anti-PD-L1	Lung cancer	IL-8	6
2	Zhao et al., 2021, (17) China	40	85	Anti-PD-1	Lung cancer	IL-5; IFN-γ	8
3	Zhang et al., 2021, (18) China	20	90	Camrelizumab	Esophageal cancer	IL-15	6
4	Wang et al., 2021, (19) America	462	62	Immune checkpoint blockade	Melanoma	IL-6	9
5	Shi et al., 2021, (20) China	60	70	Anti-PD-1-based chemotherapy	Lung cancer	IL-1β; IL-2; IL-4; IL-5; IL-6; IL-8; IL-10; IFN-γ; TNF-α	7
6	Shenderov et al., 2021, (21) America	30	NR	Nivolumab + ipilimumab	Prostate cancer	IL-1β; IL-5; IL-6; IL-8; IL-15; TNF-α	8
7	Klerk et al., 2021, (22) America	49	79.6	Pembrolizumab	Esophageal cancer	IL-6	9
8	Arends et al., 2021, (23) America	367	80.9	Durvalumab	Head and neck cancer	IL-6; IL-8	7
9	Yuen et al., 2020, (24) America	1,020	78.8	Atezolizumab	Urothelial carcinoma Renal cell carcinoma	IL-8	10
10	Schalper et al., 2020 (7), America	1,642	66.7	Nivolumab Ipilimumab Nivolumab + Ipilimumab	Melanoma Renal cell carcinoma Lung cancer	IL-8	9
11	Pedersen et al., 2020, (25) Denmark	16	69	Pembrolizumab Nivolumab + ipilimumab	Melanoma	IL-6; IL-8; IL-10; IFN-γ; TNF-α	8
12	Laino et al., 2020 (26), America	1,256	NR	Nivolumab Ipilimumab Nivolumab + Ipilimumab	Melanoma	IL-6	9
13	Koh et al., 2020 (27), Korea	132	79.5	Pembrolizumab Nivolumab	Lung cancer	IL-10	6
14	Keegan et al., 2020 (28), America	47	34	Pembrolizumab Nivolumab Atezolizumab	Lung cancer	IL-6	8
15	Kang et al., 2020 (29), Korea	125	79.2	Nivolumab Pembrolizumab Atezolizumab	Lung cancer	IL-6	7
16	Babačić et al., 2020 (30), Sweden	24	54.2	Anti-PD-1	Melanoma	IL-6; IL-12	6
17	Agulló-Ortuño, et al., 2020 (31), Spain	27	78	Nivolumab	Lung cancer	IL-8	7
18	Lim et al., 2019 (13), Australia	38	74	Anti-PD-1 Anti-CTLA-4 + anti-PD-1	Melanoma	IL-2; IL-8; TNF-α	7
19	Hardy-Werbin et al., 2019 (32), Spain	37	64.9	Ipilimumab	Lung cancer	IL-1β; IL-2; IL-4; IL-5; IL-6; IL-8; IL-10; IFN-γ; TNF-α	9
20	Valpione et al., 2018 (33), England	140	61.4	Ipilimumab	Melanoma	IL-6	8
21	Tallerico et al., 2017 (34), Italy	116	NR	Ipilimumab	Melanoma	IL-15	7
22	Sanmamed et al., 2017 (35), America	48	60.4	Nivolumab Pembrolizumab	Melanoma Lung cancer	IL-8	7
23	Damuzzo et al., 2016 (36), Italy	44	65.9	Ipilimumab	Melanoma	IL-6	8
24	Bjoern et al., 2016 (37), Denmark	40	40	Ipilimumab	Melanoma	IL-6	9

IL, interleukin; IFN, interferon; TNF, tumor necrosis factor.

### Peripheral cytokine levels and its association with OS

**Figure 2:**
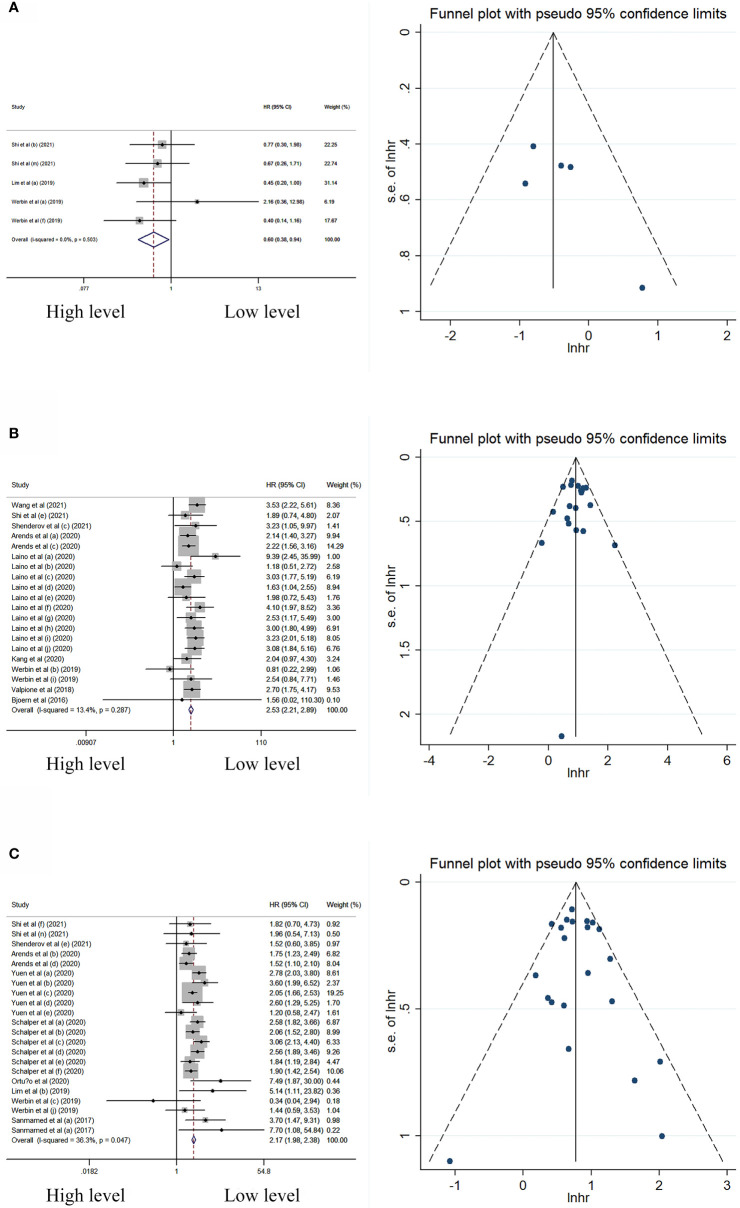
Forest plot and funnel plot of studies investigating levels of peripheral **(A)** IL-2, **(B)** IL-6, and **(C)** IL-8 in univariate analysis of OS.

In univariate analysis, peripheral IL-2 level was significantly associated with better OS, while the levels of IL-6 and IL-8 were significantly associated with worse OS (**Table 2**; **Figure 2**). Neither significant heterogeneity nor publication bias was detected in these meta-analyses.

**Table 2:** Meta-analysis investigated the association between cytokines and OS.

Cytokines	Studies (*n*)	HR (95% CI)	*p*-value	*I* ^2^ (%)	*p*-value Egger’s test
Univariate Analysis					
IL-1β	3	0.60 (0.33, 1.09)	0.09	0	0.11
**IL-2**	3	0.60 (0.38, 0.94)	0.02	0	0.14
IL-4	2	0.96 (0.49, 1.88)	0.90	49	0.12
IL-5	3	0.77 (0.44, 1.34)	0.36	0	0.17
**IL-6**	9	2.53 (2.21, 2.89)	<0.00001	13	0.89
**IL-8**	9	2.17 (1.98, 2.38)	<0.00001	36	0.61
IL-10	3	1.61 (0.91, 2.83)	0.10	0	0.32
TNF-α	4	1.44 (0.66, 3.14)	0.35	60	0.66
**Multivariable Analysis**					
**IL-6**	8	2.21 (1.67, 2.93)	<0.00001	85	0.00
**IL-8**	2	1.88 (1.70, 2.07)	<0.00001	0	0.68
**IL-15**	2	1.77 (1.08, 1.32)	0.0006	30	0.64

IL, interleukin; TNF, tumor necrosis factor.

**Figure 3:**
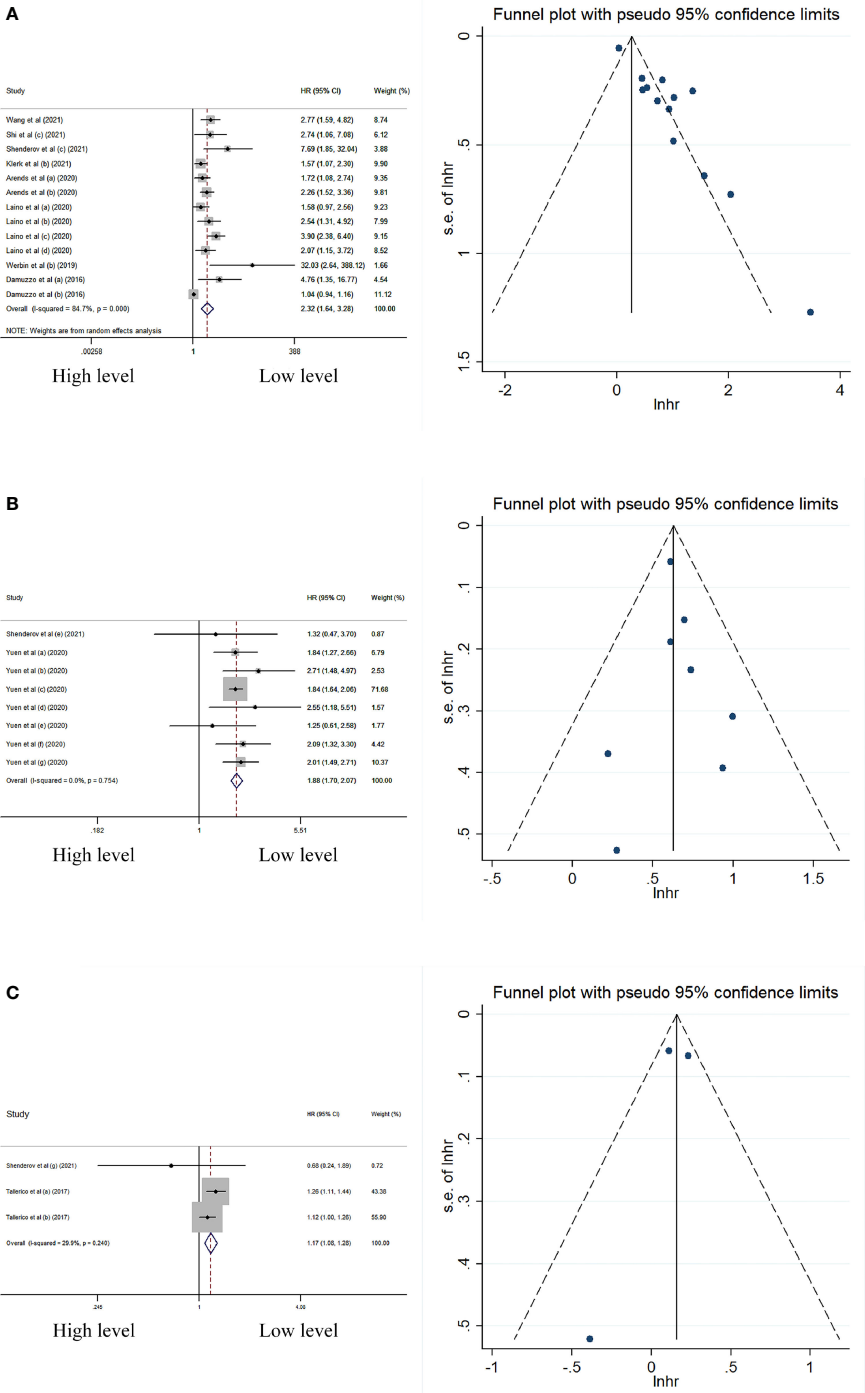
Forest plot and funnel plot of studies investigating levels of peripheral **(A)** IL-6, **(B)** IL-8, and **(C)** IL-15 in multivariable analysis of OS.

**Figure 4:**
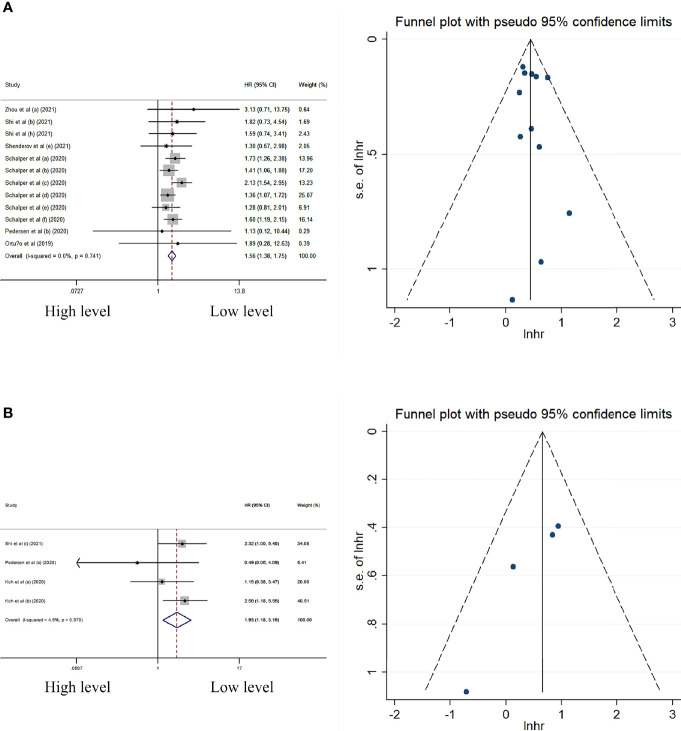
Forest plot and funnel plot of studies investigating levels of peripheral **(A)** IL-8 and **(B)** IL-10 in univariate analysis of PFS.

In multivariate analysis, the peripheral level of IL-6, IL-8, and IL-15 was significantly associated with worse OS (**Table 2**; **Figure 3**). No significant heterogeneity and publication bias were observed in summary HRs of IL-8 and IL-15. The sensitivity analyses for IL-8 and IL-15 also displayed relatively robust results (**Figures 4E, F**). However, the heterogeneity in the summary effect for IL-6 was large (*I* ^2^ = 85% [75%–91%]). Subgroup analysis showed that European patients, melanoma, ipilimumab monotherapy, blood sample type, on-treatment sample type, and using flow cytometry-based detection method were associated with higher heterogeneity compared with other regions (e.g., America or China), other cancer types (e.g., lung cancer or prostate cancer), other treatment methods (e.g., nivolumab or pembrolizumab), other sample types (serum or plasma), other detection methods (e.g., ELISA or Luminex), and baseline sample type (**Table 3**). Meta-regression analysis did not show any statistically significant covariates that could explain the heterogeneity (**Supplementary Table 3**). However, the funnel plot for IL-6 had apparent asymmetry and the *p*-value of Egger’s test was less than 0.05 (Egger’s test: *p*-value = 0), indicating the existence of publication bias.

**Table 3:** Subgroup analysis of IL-6 multivariable OS.

Subgroup	*N* studies	Hazard ratio (95% CI)	*I* ^2^ (%)	*p*-value for heterogeneity
Region				
Europe	2	3.82 (0.69,21.16)	84	0.002
America/China	6	2.24 (1.79, 2.81)	41	0.08
**Cancer type**				
Melanoma	3	2.24 (1.34, 3.74)	89	<0.00001
Other	5	2.25 (1.52, 3.35)	55	0.05
**Drug**				
Ipilimumab	5	3.16 (1.58, 6.33)	90	<0.00001
Other	3	1.87 (1.54, 2.28)	0	0.58
**Sample source**				
Blood	1	1.95 (0.45, 8.43)	82	0.02
Plasma/serum	7	2.33 (1.81, 2.99)	49	0.03
**Detection time**				
On-treatment	2	1.50 (0.60, 3.78)	75	0.04
Baseline	7	2.38 (1.83, 3.09)	52	0.02
**Detection method**				
Flow cytometry	1	1.95 (0.45, 8.43)	82	0.02
ELISA/Luminex	7	2.33 (1.81, 2.99)	49	0.03

No statistical significance was observed in the univariate analysis of IL-1β, IL-4, IL-5, IL-10, and TNF-α. The forest plots for these cytokines are shown in **Supplementary Figure 1**.

### Peripheral cytokine levels and its association with PFS

In univariate analysis, the peripheral levels of IL-8 and IL-10 were associated with better PFS without significant heterogeneity and publication bias in these meta-analyses (**Table 4**; **Figure 4**).

**Table 4:** Meta-analysis investigated the association between cytokines and PFS.

Cytokines	Studies (*n*)	HR (95%CI)	*p*-value	*I* ^2^(%)	*p*-value Egger’s test
Univariate Analysis					
IL−5	3	1.15 (0.72, 1.85)	0.56	45	0.64
IL−6	5	1.61 (0.86, 3.02)	0.1	53	0.90
**IL-8**	6	1.56 (1.38, 1.75)	<0.00001	0	0.59
**IL-10**	3	1.93 (1.18, 3.16)	0.001	5	0.06
TNF-α	3	0.71 (0.14, 3.57)	0.73	74	0.06
IFN-γ	3	1.11 (0.40, 3.07)	0.85	65	0.87
**Multivariable Analysis**					
IL-6	3	1.75 (0.43, 7.08)	0.45	88	0.29

IL, interleukin; IFN, interferon; TNF, tumor necrosis factor.

**Figure 5:**
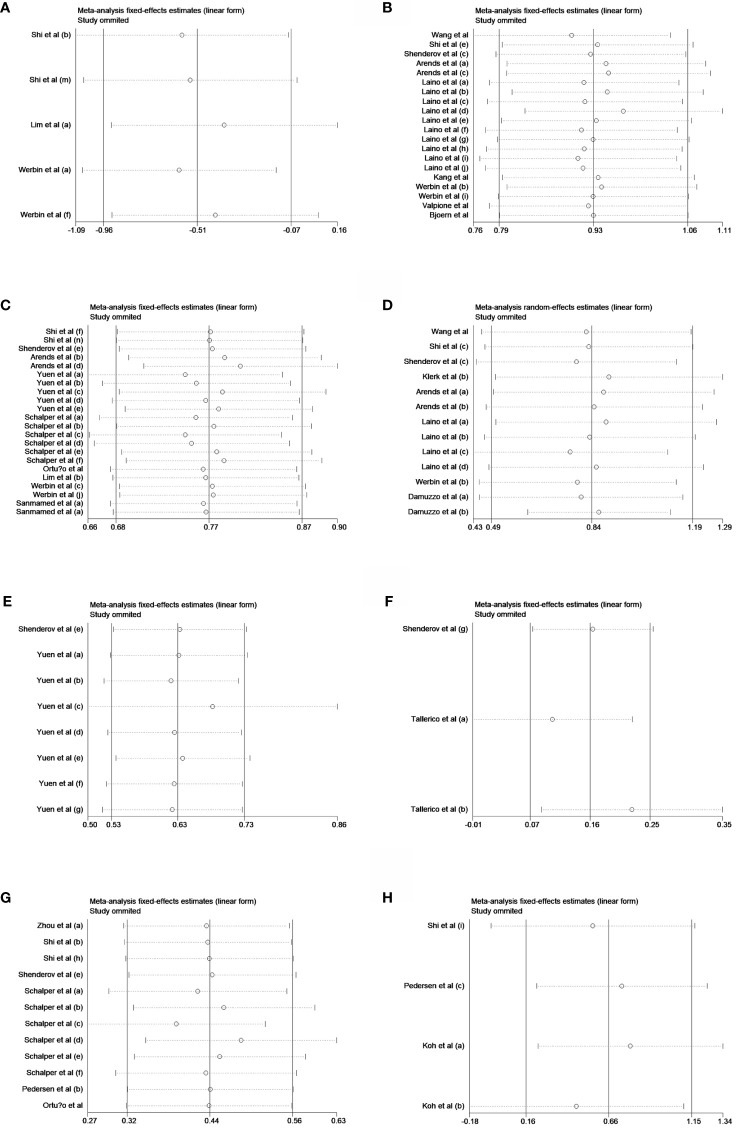
Sensitivity analyses of peripheral **(A)** IL-2, **(B)** IL-6, and **(C)** IL-8 in univariate analysis of OS and **(D)** IL-6, **(E)** IL-8, and **(F)** IL-15 in multivariable analysis of OS and **(G)** IL-8 and **(H)** IL-10 in univariate analysis of PFS.

**Figure 6:**
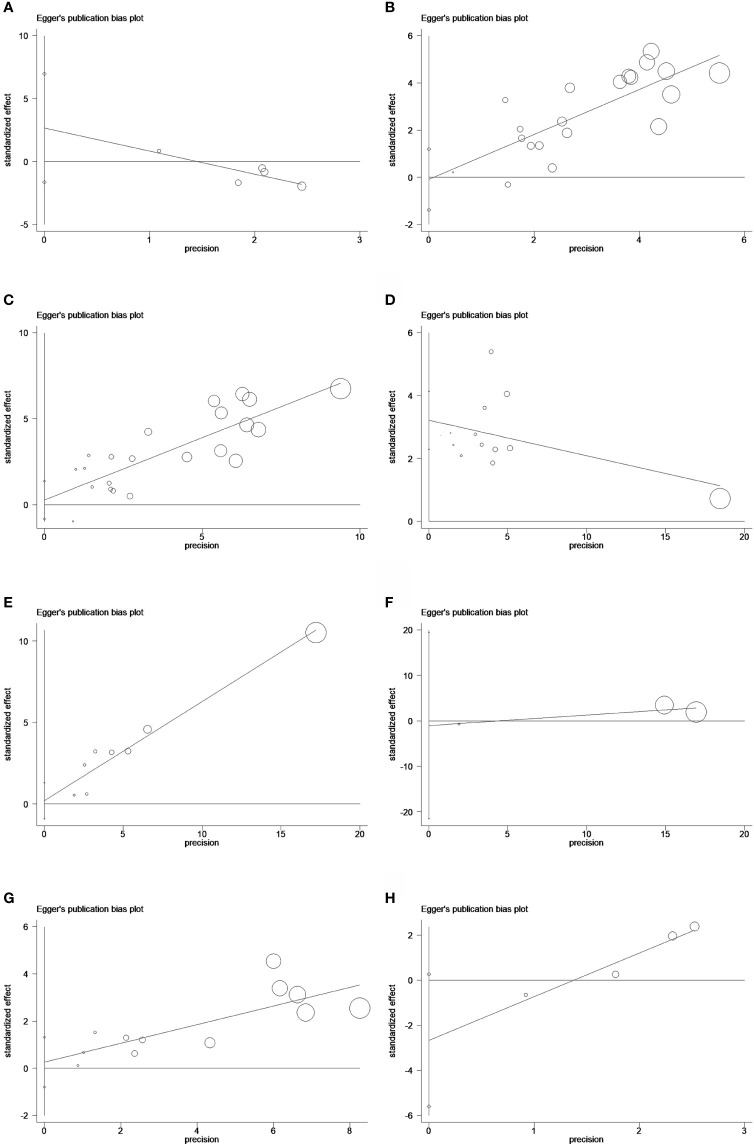
Egger’s publication bias plots of peripheral **(A)** IL-2, **(B)** IL-6, and **(C)** IL-8 in univariate analysis of OS and **(D)** IL-6, **(E)** IL-8, and **(F)** IL-15 in multivariable analysis of OS and **(G)** IL-8 and **(H)** IL-10 in univariate analysis of PFS.

No statistical significance was observed in the univariate analysis of IL-5, IL-6, TNF-α, and IFN-γ, and in the multivariable analysis of IL-6. The forest plots for these cytokines are shown in **Supplementary Figures 2, 3**.

### Sensitivity analyses and Egger's publication bias plots

Sensitivity analyses showed that all pooled results were relatively robust (**Figures 5A–H**). Egger's publication bias plots were shown in **Figures 6A–H**.

## Discussion

This meta-analysis investigated the association between the peripheral cytokine levels and the outcome of ICIs in cancers. Low levels of IL-6, IL-8, and IL-15 were found on multivariable analysis to be associated with better OS of cancer patients treated with ICIs. Although the levels of IL-2, IL-6, IL-8, and IL-10 could also affect PFS or OS of patients in univariate analysis, the statistical evidence of univariate analysis was weak and further studies were needed to confirm these findings.

To the best of our knowledge, this is the first meta-analysis to investigate how peripheral cytokines affect the efficacy of ICI treatment in cancer patients. In the univariate analysis of OS, patients treated with ICIs whose IL-2 increased had a better OS. IL-2 plays a significant role in modulating the immune system, in both innate and adaptive immunity (10). IL-2 is not only an essential mediator that promotes T-cell clonal expansion, survival, and differentiation after antigen exposure, but also a growth factor and enhancer for CD4^+^T cells as well as natural killer (NK) cells (10, 32). Because modulating the activity of T cells through the interaction between immune checkpoint molecules may lead to decreased IL-2 secretion, the release of this inhibition with ICIs could increase IL-2 secretion (38), which, in turn, enhances the immune response. However, multiple latest studies have shown that IL-2 induces immunosuppressive regulatory T (Treg) cell activity through binding with a high-affinity trimeric receptor composed of α, β, γ subunits on the surface of Treg cell and thereby inhibit antitumor response (10, 39). Given the possible double effect of IL-2 in tumor immune environment and the relatively few studies that have been published, the relationship between IL-2 and ICIs needs further study.

In the univariate analysis of PFS, high level of IL-10 was associated with worse PFS in cancer patients. IL-10 is usually considered as an immunosuppressive and anti‐inflammatory cytokine regulating the growth and differentiation of various immune cells (40). The inhibitory effect of IFN-γ secretion mediated by IL-10 was achieved *via* interacting with APC function of macrophages and inhibiting cytokines produced by activated dendritic cells (41). An additional property of IL-10 is its anti-inflammatory trait to inhibit the antigen-presenting capacity of monocytes (42). All the above were aligned with the results of our meta-analysis. However, it has been recently shown that IL-10 may stimulate the immune response on specific cell types and contexts (41). High level of IL-10 was able to promote activation and proliferation of CD8+ T cells in chronic inflammation and cancer (43). The efficacy and safety of high-dose IL-10 combined with ICIs in cancer treatment have been verified in clinical trials, and the clinical results were safe and tolerated (NCT02009449). In light of recent studies, we have to revisit our research data. While the heterogeneity was small, the survival data from two of the four studies were not statistically significant, and more data may be needed to demonstrate the prognostic significance of IL-10 in ICI treatment.

IL-15, a member of the four-α-helix bundle family of cytokines, could stimulate the proliferation of immune cells including T cell, B cell, and NK cell through JAK1/3 STAT3/5 signaling molecules (39). Combination therapy of IL-15 and ICIs was able to facilitate clearance of immune checkpoints and to achieve prolonged survival in animal experiments (44, 45). In addition, an antitumor combination strategy, IL-15 in combination with monoclonal antibody, was gradually emerging, and clinical trials for leukemia and lymphoma have been carried out (46). Taking all the above results, IL-15 seems to act as an anti-cancer factor, which is contrary to our conclusion. Given that only three survival data included in the statistical analysis of IL-15, and one of them was not statistically significant, more data were still needed to further verify this finding.

Until now, there were relatively numerous studies discussing the association with IL-6, IL-8, and ICI treatment. The statistical analysis of IL-6 and IL-8 was robust and adequate in our meta-analysis, and the result showed that high levels of IL-6 and IL-8 were significantly associated with the prognosis of cancer patients treated with ICIs, for both OS and PFS. IL-6 is emerging as a regulator of immune system through multiple mechanisms, especially in patients treated with ICIs, from promoting expression of the T helper-associated cytokine IL-4 (47) and suppressing IFN-γ expression (48), to inducing the expression of angiogenic factors, such as vascular endothelial growth factor (VEGF), and downregulating expression of CD80, CD86, and IL-12 (49). In addition to its immunosuppressive function, IL-6 also has multiple pro-tumorigenic activities. IL-6 contributes to malignancy formation and proliferation by inducing expression of STAT3 and its downstream target genes, which encode proteins that drive tumorigenesis, such as cyclin D1 or BCL2- like protein 1 (BCL-xL) (26). STAT3, in turn, promotes IL-6 gene expression, which forms a positive feedback loop to accelerate tumor formation. There have been several studies suggesting that inhibiting IL‐6 activity can increase the response rate of cancer patients to ICIs, and ultimately improve outcomes for patients (23). Combined blockade of IL-6 and PD-1/PD-L1 abolishes mutual regulation of their immunosuppressive effects and lead to synergistic antitumor effects. Recently, clinical trials of ICIs combined with anti-IL-6 or anti-IL-6 receptor for cancer patients have started or are being initiated (NCT03999749, NCT04258150, and NCT03821246) (23).

IL-8, a pro-inflammatory chemokine and overexpressed in many cancers, was shown to enhance the growth and invasion abilities of tumor cells in multiple mechanisms, including angiogenesis, epithelial-to-mesenchymal transition (EMT), formation of neutrophil extracellular traps (NETs), and infiltration of immunosuppressive and pro-tumorigenic myeloid inflammatory cells (50–52). Multiple studies have shown that the NF-kB signaling pathway plays a significant role in maintaining the mesenchymal phenotype of tumor cells and inducing cytoskeletal rearrangement to increasing chemotaxis and invasion (53, 54). On the process shown above, IL-8 binds to CXCR1 on tumor cells in an autocrine manner to trigger a downstream cascade reaction of the signaling pathway resulting in increased NF-kB activity (54). IL-8 can alter TME in indirect ways, such as increasing angiogenesis within the tumor or recruiting higher numbers of infiltrating immune suppressive cells (55). In addition, high serum level of IL-8 is associated with tumor burden and resistance to ICIs, and blocking the IL-8/IL-8R receptor axis can augment the efficacy of ICIs (7, 55, 56). The efficacy and safety of HuMax­IL­8 (an anti-IL-8 monoclonal antibody) in multiple clinical trials are being evaluated, mostly in combination with nivolumab (NCT03689699, NCT04050462, and NCT03400332). Meanwhile, IL-8 receptor antagonists, including the dual CXCR1/2 inhibitor SX682 and the CXCR2 antagonist AZD5069, are also being assessed in combination with multiple anti-PD-1 or anti-PD-L1 mAbs (55).

It is noteworthy that in addition to the above-discussed cytokines, there are several other cytokines directly involved in the activation of an effective antitumor response, such as IL-12 and TGF-β (15). Disappointingly, there were not enough studies to conduct meta-analyses for IL-12 and TGF-β after conducting systematic retrieve. There were four articles investigating the association of IL-12 and the efficacy of ICIs. Only the study of Babačić et al. (30) contained the relevant survival data, and the remaining three studies (57–59) only indicated that the higher level of IL-12 at baseline was associated with clinical benefit from ICIs; however, no relevant survival data were provided. For TGF-β, only four studies roughly met our inclusion criteria after screening title and abstracts. Two studies (60, 61) investigated gene signatures of TGF-β rather than peripheral TGF-β concentration. One study (62) measured the peripheral level of TGF-β 1 week after anti-PD-1 immunotherapy, which did not meet our requirement. The work of Feun et al. (63) provided KM curves for OS and PFS based on the baseline plasma level of TGF-β in hepatocellular carcinoma (HCC) patients treated with pembrolizumab. Relevant survival data can extract from curves, but the accuracy is poor. Therefore, more high-quality clinical studies are warranted to assess the predictive value of IL-12 and TGF-β for ICI treatment.

There are several limitations in our meta-analysis. First, this study is only based on the data of the three commonly used public databases (PubMed, Embase, and Cochrane of library), and thus relevant papers may be omitted. Second, on some occasions, we had to calculate HR and 95% CI based on the data extracted from the Kaplan–Meier survival curve, which may not provide the most accurate survival data. However, this practice did not significantly disrupt the stability of the result. Third, significant heterogeneity was observed in the multivariable analysis of evaluating the prognostic role of IL-6 for OS, which might be attributed to cancer type, treatment method, sample source, detection time, and detection method according to the results of subgroup analysis. In addition, we also perform a meta-regression analysis to discuss the source of heterogeneity; however, no statistically significant covariates were observed.

## Conclusion

Our study is the first comprehensive meta-analysis investigating the association between peripheral cytokine levels and the outcome of ICI treatment in cancer patients to date. The peripheral level of IL-8 may be used as a prognostic marker to identify patients with inferior response to ICIs. More high-quality prospective studies are warranted to assess the predictive value of peripheral cytokines for ICI treatment.

## Data availability statement

The original contributions presented in the study are included in the article/**Supplementary Material**. Further inquiries can be directed to the corresponding author.

## Author contributions

X-CM and TL designed the study. C-CY, Y-FY, and L-JY performed the systematic search. X-CM and HL selected eligible articles and conducted the quality assessment. X-CM, Z-ND, Y-CY, Z-RD, and D-XW analyzed and interpreted the data, and drafted the manuscript. TL revised the manuscript. All authors have read and approved the final version of the manuscript.

## Funding

This work was supported by grants from the Taishan Scholars Program for Young Expert of Shandong Province (Grant No. tsqn20161064), National Natural Science Foundation of China (Grant Nos. 82073200 and 81874178), Funds for Independent Cultivation of Innovative Team from Universities in Jinan (Grant No. 2020GXRC023), and Major Basic Research of Shandong Provincial Natural Science Foundation (Grant No. ZR202105070027).

## Conflict of interest

The authors declare that the research was conducted in the absence of any commercial or financial relationships that could be construed as a potential conflict of interest.

## Publisher’s note

All claims expressed in this article are solely those of the authors and do not necessarily represent those of their affiliated organizations, or those of the publisher, the editors and the reviewers. Any product that may be evaluated in this article, or claim that may be made by its manufacturer, is not guaranteed or endorsed by the publisher.

## Abbreviations

ICIs, immune checkpoint inhibitors; FDA, Food and Drug Administration; LAG-3, lymphocyte activation gene-3; TIM-3, T-cell immunoglobulin and mucin-domain containing-3; TIGIT, T-cell immunoglobulin and ITIM domain; IrAEs, immune-related adverse events; TMB, tumor mutational burden; TME, tumor microenvironment; TNF, tumor necrosis factor; OS, overall survival; PFS, progression-free survival; HR, hazard ratio; CI, confidence interval; NOS, Newcastle–Ottawa scale; NK, natural killer; Treg, regulatory T; IFN, interferon; VEGF, vascular endothelial growth factor; BCL-xL, BCL2-like protein 1; EMT, epithelial-to-mesenchymal transition; NETs, neutrophil extracellular traps; HCC, hepatocellular carcinoma.
